# Phase transitions, mechanical properties and electronic structures of novel boron phases under high-pressure: A first-principles study

**DOI:** 10.1038/srep06786

**Published:** 2014-10-27

**Authors:** Changzeng Fan, Jian Li, Limin Wang

**Affiliations:** 1 State Key Laboratory of Metastable Materials Science and Technology, Yanshan University, Qinhuangdao 066004, China

## Abstract

We have explored the mechanical properties, electronic structures and phase transition behaviors of three designed new phases for element boron from ambient condition to high-pressure of 120 GPa including (1) a *C2/c* symmetric structure (*m*-B_16_); (2) a 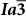 symmetric structure (*c*-B_56_) and (3) a *Pmna* symmetric structure (*o*-B_24_). The calculation of the elastic constants and phonon dispersions shows that the phases are of mechanical and dynamic stability. The *m*-B_16_ phase is found to transform into another new phase (the *o*-B_16_ phase) when pressure exceeds 68 GPa. This might offer a new synthesis strategy for *o*-B_16_ from the metastable *m*-B_16_ at low temperature under high pressure, bypassing the thermodynamically stable *γ*-B_28_. The enthalpies of the *c*-B_56_ and *o*-B_24_ phases are observed to increase with pressure. The hardness of *m*-B_16_ and *o*-B_16_ is calculated to be about 56 GPa and 61 GPa, approaching to the highest value of 61 GPa recorded for *α*-Ga-B among all available Boron phases. The electronic structures and bonding characters are analyzed according to the difference charge-density and crystal orbital Hamilton population (COHP), revealing the metallic nature of the three phases.

Boron has been recognized as a mystical and important element due to its fascinating chemical and physical properties^1^. However, most of the reported phases are likely to be boron-rich borides^2^,^3^,^4^ and probably only four phases correspond to the pure element. This might be ascribed to the extreme sensitivity of the element to even small amounts of impurities, given that there are a number of boron-rich compounds with unique icosahedral structures such as YB_66_, B_6_O, AlB_12_, B_13_P, B_50_C_2_^5^. Such a complexity may arise from its unique location in the periodic table: situates at the boundary between metals and nonmetals and is the only nonmetal element of group III elements. Extensive studies have been performed to investigate the crystal structures and phase stabilities of this element^6^,^7^,^8^,^9^,^10^,^11^,^12^. Most of the reported boron has complicated crystal structures based on icosahedral B_12_ clusters, which can be flexibly linked into rigid frameworks. Such a special structure makes the boron have unique properties among elemental materials, such as a low volatility, a high melting point (2450°C) as well as excellent mechanical properties including high strength and hardness^13^.

Now, only three of the reported boron phases have been confirmed to be thermodynamically stable, *i.e.*, *α*-rhombohedral boron (*α*-B)^14^, *β*- rhombohedral boron (*β*-B)^15^,^16^ and *γ*-B^17^,^18^. The *α*-B was identified in 1958^19^, featured by a single icosahedron in a slightly distorted cubic cell with the C_3_ axis of the icosahedron aligned to the *c*-axis of the unit-cell^20^. In 1957, Sands and Hoard announced the identification of the *β*-B^21^. Experiments^9^,^22^ and theories^23^,^24^ are thereafter carried out and tested for this phase. Shirai *et.al.*^25^,^26^,^27^ studied diagram of *α*-B and *β*-B and found that at zero temperature *α*-B is more stable than *β*-B. However, the stability of these two phases remains uncertain at low pressure. Oganov *et al*.^5^,^8^,^18^ reported an ionic phase of element boron (*γ*-B), which consists of icosahedral B_12_ clusters and interstitial B_2_ pairs acting as anions and cations, respectively, in a NaCl-type arrangement. The ionicities of boron-boron bonds in B_12_ icosahedra of *α*-B have also been reported in one of our previous work^28^.

In addition, other phases have also been reported. The first crystal structure of *α*-tetragonal boron called T-50 was identified in 1951^29^, constituted by 50 atoms in the unit cell. It was proposed that *β*-B is stable up to 30 GPa and 3500 K, and at higher temperatures and pressures, a phase transition to the tetragonal ‘T-192’ structure occurs^30^. Recently, Pickard *et al*.^8^ obtained a new metastable phase by an *ab initio* random structure searching method and this new phase can be viewed as a polymorph of *α*-B, differing in the connectivity of the icosahedral units and just 0.01 eV/atom less stable. He *et al.*^31^ subsequently studied the properties of this phase (termed α*-B) by first-principles calculations. Also, the α-Ga structure and its variants of boron also stimulate great interest in probing the high-pressure phase^5^,^18^,^32^.

In this work, we conceived three new phases with or without icosahedral unit of elemental boron. The crystal structure, phase stability, mechanical and electronic properties of these phases have been systematically studied under high-pressure based on the first-principles method.

## Results

### Structural features

**Figure 1:**
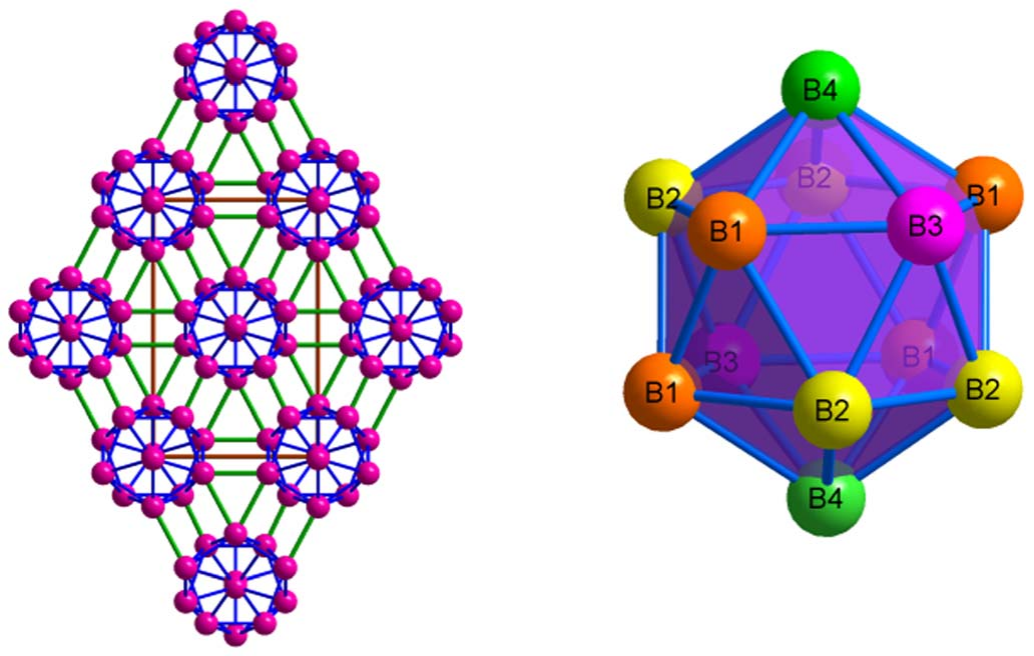
Schematic crystal structure of *o-*B_24_. B_12_ icosahedral layers viewed along [010] (Left); the icosahedral B_12_ cluster unit in the *o-*B_24_ structure (right). B_1_, B_2_, B_3_, B_4_ atom are depicted in orange, yellow, rose red, and green, respectively.

The schematic ambient crystal structure of the first new phase is shown in Figure 1, suggesting an orthorhombic structure with the space group *Pmna*. The unit cell is composed of 24 atoms, which can be classified into 4 non-equivalent atomic sites stated as B1, B2, B3, and B4. For the convenience of further discussion, we refer to it as *ο* -B_24_. Structurally, *ο* -B_24_ is another phase of boron consisting of pure slightly distorted icosahedral B_12_ clusters (except *α*-boron and α*-boron). The B_12_ cluster units are equivalent, built by 4 B1 atoms, 4 B2 atoms, 2 B3 atoms, and 2 B4 atoms. The centres of the B_12_ icosahedra projected along the [010] direction (overlapped by B4 sites) form a rhombic packing−see Figure 1. In *ο* -B_24_, the four inequivalent boron atoms can be divided into two types: the B2 and B4 atoms have five intra-icosahedral B-B bonds and another single inter-icosahedral B-B bond while the B1 and B3 atoms possess five intra-icosahedral B-B bonds and double inter-icosahedral B-B bonds. See Table S1 in the Supporting Information for more details. The relation between intra-icosahedral and inter-icosahedral bond has been intensively studied. For example, it has been generally thought that the inter-icosahedral bonding is characterized as the covalent bond and is stronger than the intra-icosahedral bonding since Longuet-Higgins disclosed the nature of the icosahedral bonding of bond molecules^33^. However, apparent contradictions have been found between such basic bonding concepts and experimental results, which may be solved by considering the special geometrical effects, as introduced to explain the elastic responses of boron carbides^34^. In table S1, we found that the shortest bonds are inter-icosahedral B_2_-B_2_ bonds (1.65 Å), indicating they are stronger than each intra-icosahedral B-B bonds. There are also some weak inter-icosahedral bonds, like B_1_-B_1_ (1.92 Å) and B_1_-B_3_ (2.04 Å), as well as some inter-icosahedral bonds comparable to those intra-icosahedral bonds, like B_4_-B_4_ (1.70 Å). To summarize, the relation between intra-icosahedral and inter-icosahedral bonds in the *ο* -B_24_ phase is quite complicated, implying it may have some special elastic responses. In addition, the coordination number *N_c_* of a selected boron atom (defined by the number of surrounding bonds with a bond length less than 2 Å) has also been investigated in this work. For *ο* -B_24_, a coordination description of *B*1^[6]^*B*2^[6]^*B*3^[5]^*B*4^[6]^ giving an average coordination numbers 5.8. In addition, each icosahedral B_12_ cluster is surrounded by eight neighbouring B_12_ clusters connected by different B-B bonds. Like α*-boron, *ο* -B_24_ may also be considered as another twinned polymorph of *α*-boron which contains pure icosahedral B_12_ clusters without linking atoms. The slightly difference in the atomic positions within an icosahedral B_12_ cluster and the difference in the packing way among B_12_ clusters in *α*-boron, α*-boron, and *ο* -B_24_ might explain their distinct symmetries. It is noted that two other known phases (*γ* -B and *β* -B) of boron also contain icosahedral B_12_ clusters. In *γ*-B, each icosahedral B_12_ cluster connects to ten equivalent neighbouring B_12_ clusters directly with inter-B_12_ B-B bonds and two secondly adjacent B_12_ clusters indirectly by interstitial B_2_ pairs^31^. The connecting between icosahedral B_12_ clusters and interstitial atoms in the *β*-B phase is more complicated.

**Figure 2:**
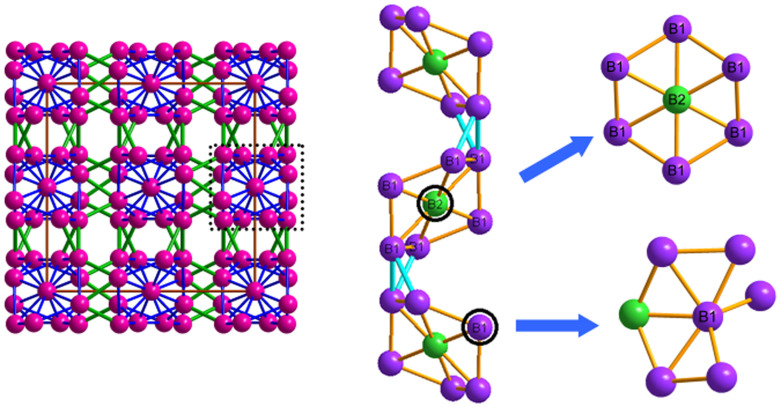
Schematic crystal structure of *c-*B_56_. Projection of the *c-*B_56_ structure along the [100] direction (left); the cubic area in the dashed box projected along the [010] direction as well as atomic environments around B1 and B2 atom (right).

The second new structure−denoted as *c*-B_56_−belongs to cubic crystal system and contains 56 atoms in the conventional unit cell (space group 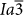, No. 206). In this structure, the ordered B atoms are clearly split into two categories, B1 and B2, which occupy Wyckoff 48*e* and 8*a* positions, respectively. The B1 atoms form a cubic area and the B2 atoms occupy its centre when the structure is projected along the [100] direction, which is denoted by the dashed box shown in the left panel of Fig. 2. The middle panel of Fig. 2 is the dashed box view along [010] direction. One can find that each B1 atom is coordinated by one B2 and five B1 atoms and each B2 atom by six B1 atoms (forming a corrugated hexagon) − see the right panel of Fig. 2. In short, the repeated cubic unit denoted by the dashed box in Fig. 2 can be considered as stacking congruent distorted hexagons linked by 2 types of B1-B1 bonds with bond lengths of 1.70 Å (B_1_-B_1_) and 1.76 Å (B_1_-B_1_′), see Table S2. There is another type of B1-B1 bond length with 1.82 Å (B_1_-B_1_″), forming the edge of the distorted hexagon. In the unit cell, one find the central three B2 atoms are bonded with surrounding twelve B1 atoms with a single bond length of 1.74 Å. Regarding the inter-cubic areas, only two types of B1-B1 bonds of 1.70 Å (B_1_-B_1_) and 1.76 Å (B_1_-B_1_′), are found to connect them of each other, see Table S2 in the Supporting Information for details.

**Figure 3:**
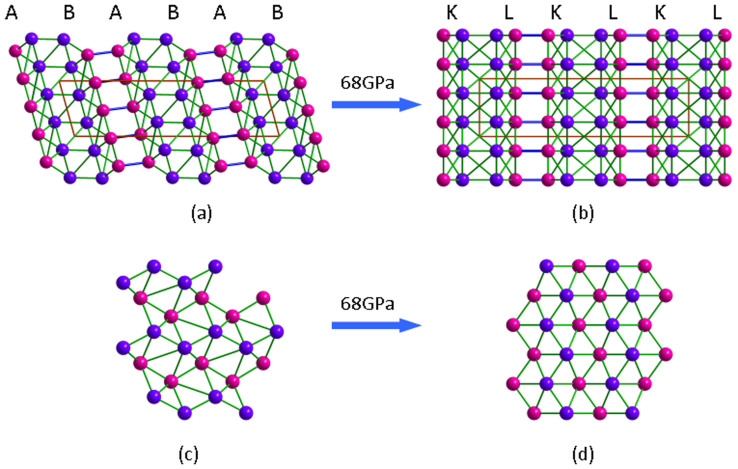
Projections of *m-*B_16_ and o-B_16_ along the [010] and [001] directions, respectively [(a) and (b)]. Projections of A layer of the *m-*B_16_ phase and K layer of the o-B_16_ phase along the [100] direction [(c) and (d)]. Purple spheres are B1 atoms, red spheres are B2 atoms.

The third new phase is a monoclinic structure that contains 16 atoms in the unit cell (space group *C2/c*, No. 15, hereafter denoted by *m*-B_16_). B atoms have two inequivalent sites in this structure, both of which occupy Wyckoff 8*f* positions. The *m*-B_16_ structure is constructed by two layers, named *A* and *B* in Figure 3 (a). The *A* layer can be viewed as strongly puckered networks connected by the different types of B-B bonds along the [100] direction (Figure 3(c)). The *B* layer is similar to *A* layer except that B1 and B2 atoms interchange their position along the [100] direction. The B-B bonds information for the A and B layers are illustrated in Table S3 in the Supporting Information. From Table S3 and Fig. 3, the A layer is bonded to B layer by two different B1-B1 bonds with bond length of 1.73 Å and 2.13 Å, respectively, while only one type of B2-B2 bond with bond length of 1.78 Å between B layer and the next A layer. It has also been found there are six different types of intra-layer bonds from Table S3. In short, each B1 atom is bonded with four B1 atoms and four B2 atoms (seven types of B-B bonds) while each B2 atom is bonded with four B1 atoms and three B2 atoms (six types of B-B bonds). Moreover, the average coordination number is calculated to be 6.0 in the *m*-B_16_ phase according to the aforementioned definition. Interestingly, when the pressure exceeds 68 GPa, the *m*-B_16_ phase transforms into another new phase. The new phase−denoted as *o*-B_16_ − belongs to orthorhombic crystal system (space group *Imma*, No. 74, see Figure 3b) and also contains 16 atoms in the unit cell. In this structure, the B atoms are also packed in a layer structure with the stacking order of *KLKLKL*… along the crystallographic *c* axis. Here both *K* and *L* denote the hexagonal boron networks, i.e., the puckered networks of titled hexagon layers by the different B atoms (B1 and B2 atoms) along *a* axis − see Figure 3 (d). Compared to the *m*-B_16_ phase, each B1 atom is bonded with the surrounding five B1 atoms and four B2 atoms (forming six types of B-B bonds) while each B2 atom is bonded with four B1 atoms and three B2 atoms (forming five types of B-B bonds) in the *o*-B_16_ phase. The detailed information can be found in Table S4 in the Supporting Information. In addition, the average coordination numbers are transformed from 6.0 in the initial *m*-B_16_ phase to 7.0 in the new *o*-B_16_ phase. One might expect the *o*-B_16_ phase has more excellent compressibility than that of *m*-B_16_ phase. The detailed optimized crystallographic data of the above boron phases can be found in Tables 1,2,3,4.

Referring to the optimized geometry crystal structures at ambient pressure, *ο* -B_24_ shows the lowest density, *ρ* = 2.22 g/cm^3^. The *c*-B_56_ is only about 2.3% denser (*ρ* = 2.27 g/cm^3^) than that of *ο* -B_24_. The density of *m*-B_16_ (*ρ* = 2.71 g/cm^3^) is higher than that of both *ο* -B_24_ and *c*-B_56_, about 22.1% and 19.4%, respectively. The *o*-B_16_ then is the phase with the highest density. With *ρ* = 2.79 g/cm^3^ it is about 3.0% denser than *m*-B_16_, 25.7% denser than *ο* -B_24_, and 22.9% denser than *c*-B_56_.

### Phase transformations

**Figure 4:**
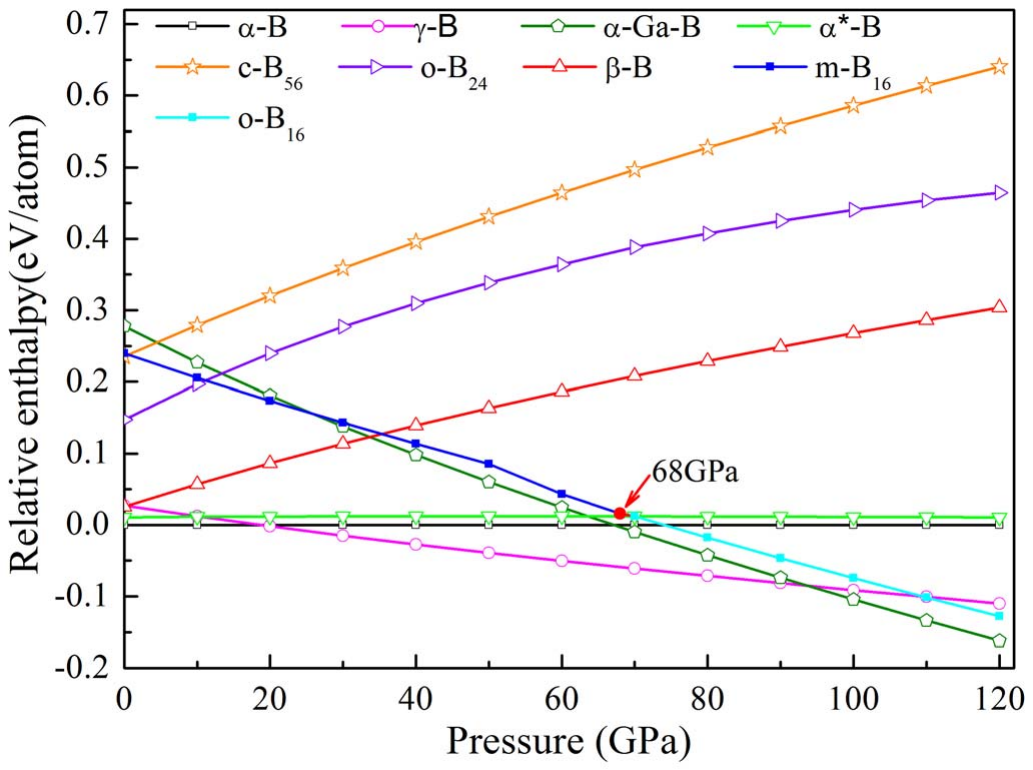
Calculated enthalpy curves (relative to *α*-boron) as a function of pressure for various boron phases.

According to our first-principles calculation results, the relative enthalpies for the chosen structures compared to that of *α*-B as a function of pressure up to 120 GPa are presented in Figure 4. It is confirmed that the *α*-B is more favorable than any other boron phases at ambient pressure. The relative enthalpy of α*-B has a constant trend within the pressure range studied, that is, the enthalpy of *α*-B is only about 0.01 eV/atom lower than that of α*-B during the whole pressure range. When pressure increases above 19 GPa, the *γ* -B has a lower enthalpy than that of *α*-B and becomes stable. The *γ* -B keeps stable up to 93 GPa and then the *α*-Ga-B prevails. Our prediction results are in accordance with the earlier study results^18^,^31^. The present calculation also shows that the enthalpy curve of the *m*-B_16_ phase under high-pressure has a similar trend as that of *α*-Ga-B and two curves intersect at about 30 GPa. In addition, it can be seen that a *m*-B_16_ → *o*-B_16_ transition occurs at 68 GPa, resulting in the enthalpy curve is slightly twisted in the range of 50 ~ 70 GPa. On the basis of Figure 4, the enthalpy curve of *m*-B_16_ and *o*-B_16_ phases are marked with blue and cyan lines, respectively. The *o*-B_16_ gets a lower enthalpy than that of *α*-B and *γ* -B at above 73 GPa and 110 GPa, respectively, implying a more stable structure than *α*-B and *γ* -B beyond the critical pressure scales. The enthalpies of the other two proposed phases, *o*-B_24_ and *c*-B_56_, increase with external hydro-pressure (like that of *β* -B), indicating that they are not favorable in the present pressure range studied.

It has been widely known that pressure and temperature are both pivotal factors that determine the states of materials. During past decades, the pressure limit for laboratory experiments has been progressively enhanced. In 1978, the pressure in the experiments with diamond−window pressure cell exceeded 178 GPa^35^. In 1986, a diamond−anvil, high-pressure apparatus was used to extend the upper limit from 210 to 550 GPa^36^, which is beyond the maximum pressure (360 GPa) of the earth′s core. In present work, pressure as high as 500 GPa have been applied in predicting the stability region of the c-B_56_ and o-B_24_ phases, unfortunately, no new stable phases have been found. We also try to investigate the high temperature stabilities of the new phases by performing quasiharmonic free energy calculations. As showed in Figure S4 of the supplementary file, it is found that the *m*-B_16_ phase transforms to the *o*-B_24_ phase when the temperature up to 575 K at ambient pressure, suggesting temperature plays a very important role during phase transition. However, in the case of *o*-B_24_, there are 200 phonon dispersions are required for a pressure point, such computational resources are not available in our group now, and will be explored in the future work.

**Figure 5:**
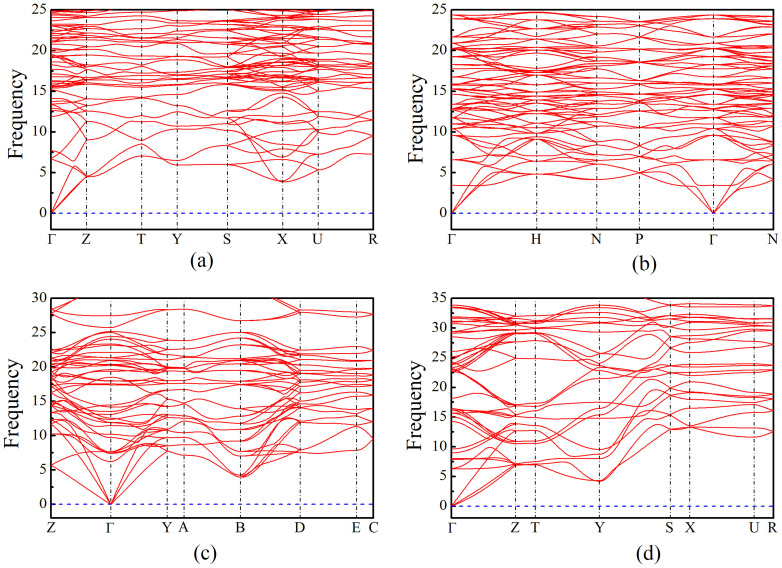
Phonon dispersion curves for the newly discovered boron phases. (a) *o*-B_24_ at 0 GPa. (b) *c*-B_56_ at 0 GPa. (c) *m*-B_16_ at 0 GPa. (d) *o*-B_16_ at 0 GPa.

To study the dynamical stability of the new high-pressure phases of boron, the phonon properties are investigated by phonon package^37^. The calculated phonon dispersion curves are shown in Fig. 5 (a)~(d), respectively. We can see that there are no imaginary frequencies for them, indicating that they are all dynamically stable.

### Mechanical properties

Several fundamental solid-state properties, such as equation of state (EOS), specific heat, thermal expansion, Debye temperature, Grüneisen parameter, melting point and many others are closely related to elastic properties of solids and, thus, the knowledge of elastic constants *C_ij_* is essential for investigating the mechanical and thermodynamic properties of a system. In calculating the elastic constants, different types of deformations are adopted for different phases according to the space group symmetry as implanted in the CASTEP code^38^. Table 5 summarizes the *C_ij_* for the new boron phases. There are three, nine or thirteen independent elastic constants for the new cubic, orthorhombic and monoclinic crystal systems, respectively. The criteria for mechanical stability^39^ of cubic phases are given by 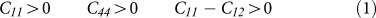
For the orthorhombic crystal, the corresponding mechanical stability criterion is as follow: 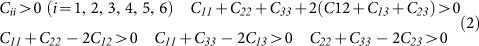
For the monoclinic structure, the mechanical stability under ambient pressure can be judged by 
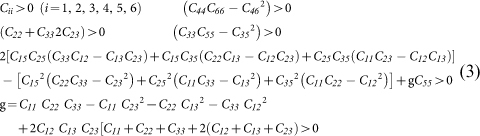
According to the above criteria, the results show that *c*-B_56_, *o*-B_24_ and *m*-B_16_ are all mechanically stable under the ambient condition.

To verify the accuracy of our calculated *C_ij_*, we fitted the first-principles calculated total energies of different boron structures at 13 different volumes to a third-order Birth-Murnaghan equation of state^40^

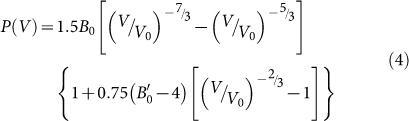
where *V_0_* is the volume per formula unit at ambient pressure, *V* is the volume per formula unit at pressure *P* given in GPa, *B_0_* is the isothermal bulk modulus, and *B_0_′* is the first pressure derivative of the bulk modulus. The values of the bulk modulus *B_0_* and its pressure derivative *B_0_′* are listed in Table 6. The bulk modulus value is in excellent agreement with that calculated from elastic constants, confirming the reliability of our calculations.

**Figure 6:**
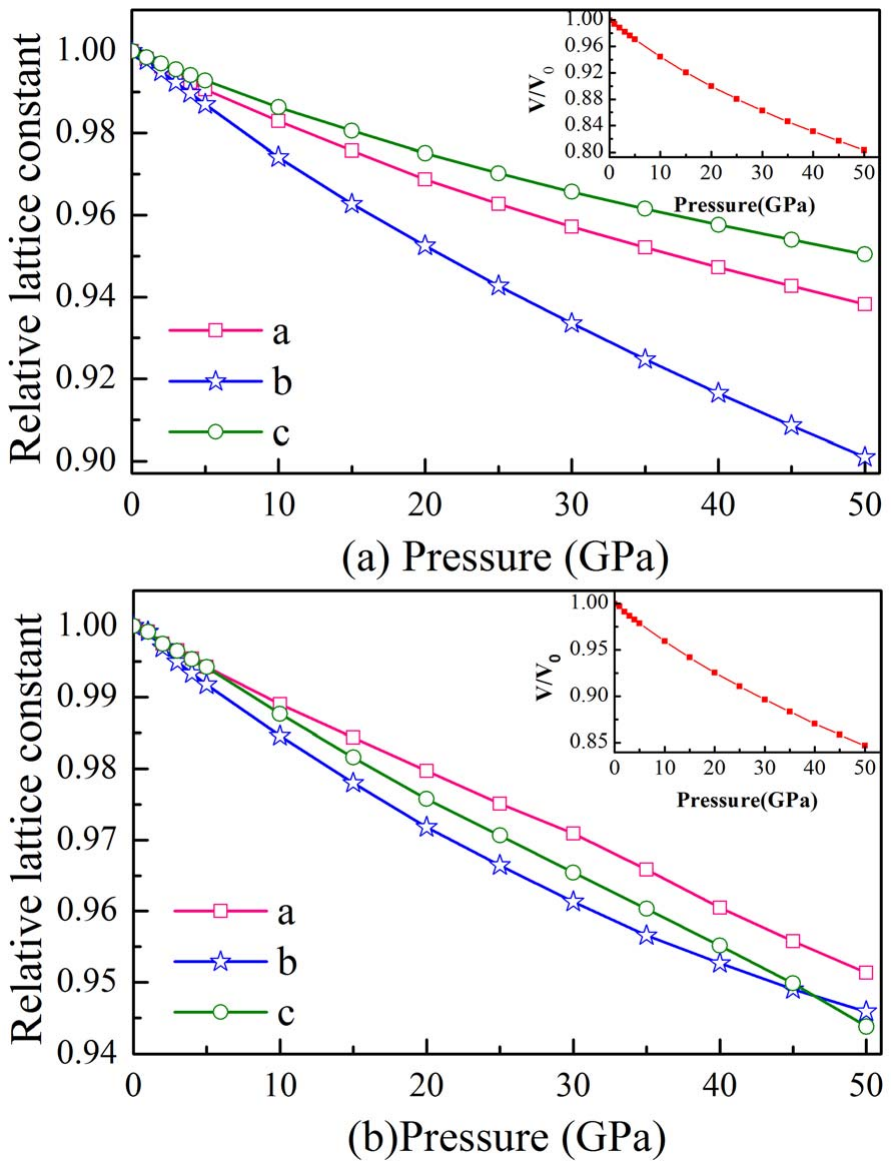
Pressure dependence of lattice constants *a*, *b*, and *c* for (a) *o*-B_24_ and (b) *m*-B_16_. The pressure dependence of cell volume is also shown in the inset.

Figure 6 (a) and (b) plot the pressure dependence of the lattice constants *a*, *b*, and *c* for *o*-B_24_ and *m*-B_16_ up to 50 GPa. It can be seen that the *b* axis is the most compressible crystallographic direction for both structures. One can also notice that the pressure has an even stronger effect on the *b* axis direction of *o*-B_24_ than on that of *m*-B_16_. Concerning the *o*-B_24_, the *c* axis is the most incompressible crystallographic direction and followed by the *a* axis. Whereas in the *m*-B_16_ structure, *a* and *c* axes exhibit similar compressibility at relatively lower pressure, the *c* axis becomes rigid with a lower compressibility than *a* axis as the pressure further increases. Interestingly, the *c* axis direction has an even lower compressibility than the *b* axis at over 47 GPa. The pressure dependence of cell volume is also shown in the insets. Comparatively, the rate of the volume shrinkage for *o*-B_24_ (about 19.7%) is larger than that of *m*-B_16_ and *c*-B_56_ (by 15.3% and 16.5%, respectively), resulting in a remarkably smaller bulk modulus than those of other two new structures (see Table 6).

Based on the Voigt-Reuss-Hill approximation^41^, the corresponding bulk and shear moduli (*B* and *G*) are obtained from the calculated elastic constants. The Young's modulus (*E*) and the Poisson's ratio (*σ*) can also be calculated from *B* and *G*. The values of *B*, *G*, *E*, *σ* and *B*/*G* for the new and known boron phases are all illustrated in Table 6. The bulk modulus is a measure of the resistance against volume change imposed by the applied pressure, while the shear modulus denotes the resistance against the reversible deformations upon shear stress^42^. The calculated shear modulus for *α*-Ga-B (297 GPa) is higher than the others, indicating it can withstand higher shear stress than other structures, followed by the new phase (*o*-B_16_) with a shear modulus of 287 GPa. On the contrary, the *o-*B_24_ phase has the lowest shear modulus. Young's modulus is a measure of the stiffness of the solid. From Table 6, the *α*-Ga-B is also of the stiffest structure, followed by *o*-B_16_. According to Ref. 42, a high (low) *B*/*G* value is associated with ductility (brittleness), and the critical value which separates ductile and brittle materials is about 1.75. In terms of the calculated values, all the studied boron phases are brittle.

The best estimates of the hardness of *α*-B and *β*-B are 42 GPa and 45 GPa^43^,^44^, respectively. According to the measured data, *γ*-B has the highest hardness (~50 GPa) of all known crystalline modifications of boron^7^. The predicted values of Vicker′s hardness for *α*-B and α*-B are 38.96 GPa and 36.60 GPa^31^. A superhard semiconducting optically transparent high pressure phase of boron with Vickers hardness of 58 GPa has also been reported^45^. The polycrystalline shear modulus is another predictor of hardness^46^, and boron suboxide (B_6_O) is a well-known superhard material with a Vicker′s hardness up to 45 GPa^47^. Compared with B_6_O (*B* = 222 GPa, *G* = 204 GPa)^48^,^49^, *m*-B_16_ has a very similar bulk modulus, but its shear modulus is 26.5% higher. Therefore, it is conceivable that *m*-B_16_ is also a superhard material considering its higher shear modulus than that of B_6_O. Thus we analyzed the hardness of different phases by adopting the empirical scheme^50^ which correlates the Vicker′s hardness and the Pugh′s modulus ratio via the formula 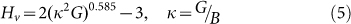
According to Eq. (5), the obtained values of Vicker′s hardness are illustrated in Table 7. From the data, *m*-B_16_ has a hardness of 56.2 GPa, which is higher than the hardness of other boron phases except for that of *α*-Ga-B^51^. Note that the hardness of the orthorhombic o-B_16_ is 60.7 GPa, suggesting the high-pressure phase transition may result in high hardness values. To give a range of possible values, we also use the Lyakhov-Oganov method^52^ to evaluate the hardness of the whole boron phases. From Table 7, one can find that there is about 20 ~ 30% difference between the calculated hardness values from the two approaches. Interestingly, we find that both *m*-B_16_ and *o*-B_16_ as well as *α*-Ga-B are potential superhard materials. Our results are in good agreement with existing experimental and theoretical data in relevant references as shown in Table 7.

### Electronic properties

**Figure 7:**
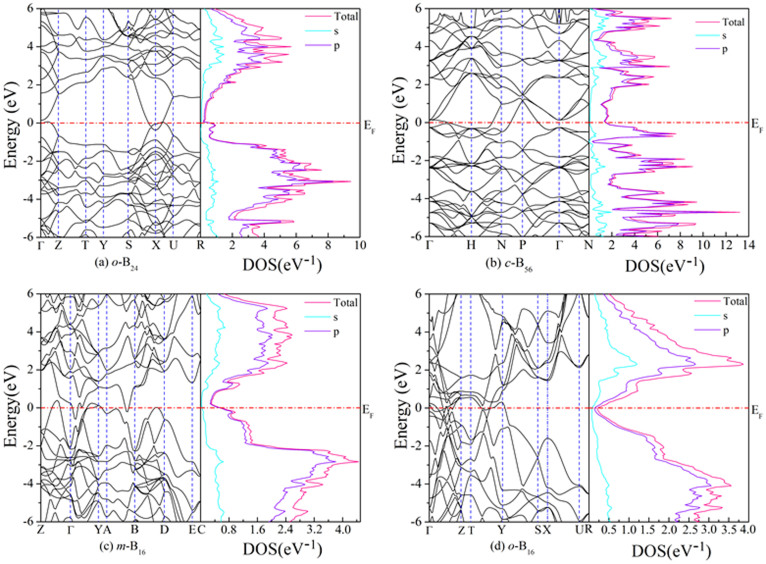
Calculated band structure along the selected high-symmetry lines and electronic DOS plots for various boron phases.

To probe the electronic properties of the four boron phases, we calculated the electronic band structure and the partial electronic density of states (DOS) for *o*-B_24_, *c*-B_56_, and *m*-B_16_ at 0 GPa and *o*-B_16_ at 68 GPa, as shown in Figure 7. It can be clearly seen that the four phases are metallic as the energy bands crossing over the Fermi level (E_F_), which are quite different from most known boron phases. It is generally known that boron has many hypothetical structures, and some of them are also predicted to be metallic^53^,^54^,^55^. However, none of these phases have been confirmed experimentally. Many scholars have made unremitting efforts to understand difficulties of boron crystals by the experimental and theoretical methods. Such difficulties maybe attributed to the defect states, which are found commonly in complicated boron crystals^24^,^56^,^57^. According to Ref. 57, boron crystals show another freedom − compositional freedom for complicated crystals, which causes deviation from stoichiometry, such effect has also been found in boron compounds like BeB_2_^58^,^59^,^60^,^61^,^62^. A possible consequence is that doping is countered by change of defect density in a way that the system is kept semiconducting, such as the known *β*-phase. In case of proposed structures, since the host structures are band crossing metal, above scenario does not apply. Instead, introduction of defects may lead to the shift Fermi level in a way that density states at the Fermi level are minimized.

The total electronic DOS at the Fermi level for the *m*-B_16_ and *o*-B_16_ phases is 0.55 and 0.21 eV^−1^, respectively, both of which are smaller than that (0.64 and 1.49 eV^−1^, respectively) of the *o*-B_24_ and c-B_56_ phases. Moreover, we can see that both conduction and valence band for the whole phases are contributed from the *p* states, especially around the Fermi level.

**Figure 8:**
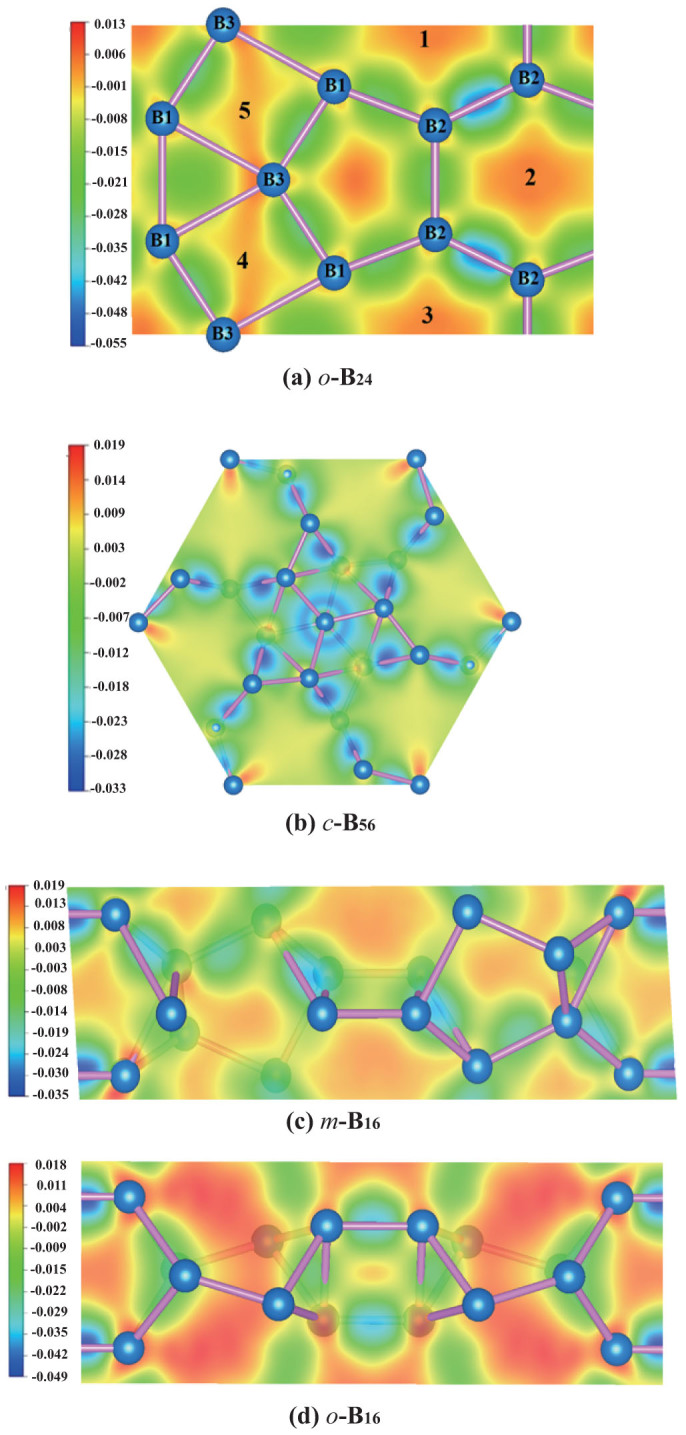
Difference charge-density distribution maps for the selected slices of (a) *o*-B_24_, (b) *c*-B_56_, (c) *m*-B_16_ and (d) *o*-B_16_.

Figure 8 plots the difference charge density maps of the four new structures along selected planes at ambient pressure. In the present work, the charge density difference is defined as: 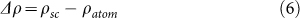
where *ρ_sc_* is the surface charge density obtained after self-consistent calculations. *ρ_atom_* is the surface charge density obtained by corresponding non-self-consistent calculations, namely, it is a superposition of atomic charge densities of the original geometry configuration. Therefore, the charge density difference maps can be used to analyze the charge transfer before and after the electronic structure relaxation as well as the charge transfer during the corresponding bonds forming procedure. Before carrying out such calculations, the validity of our calculation procedure has been confirmed by reproducing the differential charge density maps as shown in Fig. 4 of Ref. 31.

For the convenience of discussion, the locations of boron atoms are also marked. For the phase *o*-B_24_ containing icosahedra, one observes that there are electrons distribute on the centre of boron pentagon−see the centre of Figure 8 (a), indicating that the charge accumulation occurs inside the icosahedral B_12_ units. It is obvious that a smaller charge accumulation around the B atoms (the pentagon forming ones), and some charges move from adjacent atoms forming the pentagon (the green area) to the center of the pentagon (the red area). It is worth mentioning that the nearest B-B distance is 1.65 Å, which is also the nearest distance between two adjacent icosahedra. Significant charge depletion (about 0.045 e/Å^3^) between the shortest B-B bonds in the differential charge density maps has been observed, revealing some charges are moved out of the bonds during relaxation. Such results contradict to those of *α*-Boron and α*-Boron, where the bonding electrons prefer to distribute on the inter-B_12_ B-B bonds. Such abnormal electronic response of the *ο* -B_24_ phase may also be attributed to the complicated relation between intra-icosahedral and inter-icosahedral bonds as we discussed before. In addition, some high charge accumulation in the interstitial space (marked by 1 ~ 5 in Fig. 8(a)) among icosahedra has been observed, implying gaining electrons during bonding therein.

For the *c*-B_56_ structure, the charge density difference on the (111) atomic plane is obtained. The charge depletion regions form regular hexagons, indicating the neighbouring B atoms perpendicular to the plane is losing electrons during relaxation. Compared to the other B atoms, the B atoms located on the edge of the slice show substantial charge accumulation. A small amount of electron distribution in the interstitial sites around the centre of this map can be also observed.

For the *m*-B_16_ and *o*-B_16_ structures, the charge density difference maps for the same slices are obtained as shown in Figure 8 (c) and (d). It shows that for *o*-B_16_ the electrons are gathering into the interstitial sites of lattices and surrounding the B atoms, which are more remarkable than that of *m*-B_16_. The observed accumulations of electrons may account for the stability of *o*-B_16_ at high pressure than that of *m*-B_16_.

**Figure 9:**
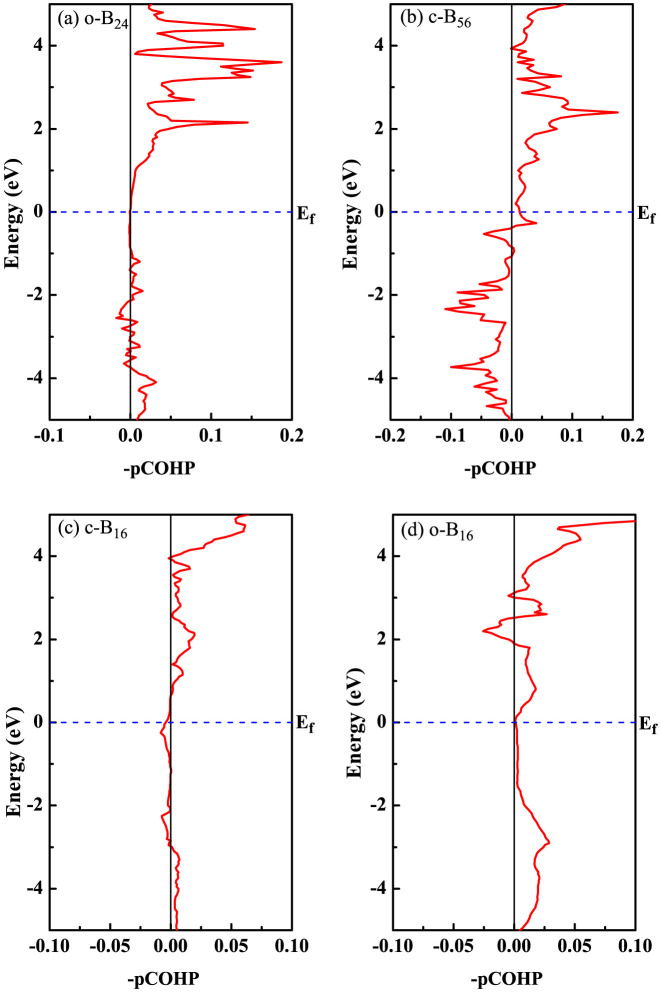
Crystal orbital Hamilton population (pCOHP) analysis for the new boron phases obtained with the PW/PAW-based method. (a) *o*-B_24_; (b) *c*-B_56_; (c) *m*-B_16_ and (d) *o*-B_16_.

Some techniques such as the crystal orbital overlap population (COOP)^63^,^64^ and its analogous crystal orbital Hamilton population (COHP)^65^,^66^ can provide a straightforward view for oribital-pair interactions. Also, based on the techniques, one can analyze and interpret the bonding situation in solid-state materials. To elucidate the bonding situation in these four new boron phases, we also perform crystal orbital Hamilton population (COHP) analysis, which is a bond-detecting tool for solids and molecules. COHP partitions the band structure energy (in term of the orbital pair contributions) into bonding, nonbonding and antibonding energy regions within a specified energy range. In Figure 9 (a)~(d), we plot –pCOHP as a function of energy for the new four phases. Positive values of –pCOHP describe bonding energy regions whereas negative values describe antibonding energy regions. As seen from the COHP diagrams in Figure 9 (a) and (b), one can observe a different picture for B-B bonding where the *o*-B_24_ phase has stronger bonds than that of the *c*-B_56_ phase. More strikingly, there appear obvious antibonding states for the *c*-B_56_ phase below *E_f_* while it is not the case for the *o*-B_24_ phase. This is in accordance to the fact that the *o*-B_24_ phase has a lower enthalpy than that of the *c*-B_56_ phase (about 0.1 eV/atom, as shown in Fig. 4). In addition, the calculated pCOHP patterns for the *m*-B_16_ and *o*-B_16_ phase bonding are not quite similar (see figure 9 (c) and (d)), suggesting that the interaction changes somehow during the *m*-B_16_ phase transforming to the *o*-B_16_ phase.

## Discussion

In this work, we extensively studied the mechanical properties, electronic structures and phase transition behaviors of three manually built new structures of element boron with (*o*-B_24_) and without (*c*-B_56_ and *m*-B_16_) icosahedra. The three new phases are proven to be mechanically and dynamically stable by computing their elastic constants and phonon dispersions. The enthalpy curve of the *m*-B_16_ phase under high-pressure has a similar trend as that of *α*-Ga-B and two curves intersect at about 30 GPa. It is found that the *m*-B_16_ phase may transform into another new phase (*o*-B_16_ with *Imma* symmetry) when the pressure reaches 68 GPa and then has a lower enthalpy than that of *α*-B beyond 73 GPa. The enthalpies of *c*-B_56_ and the *o*-B_24_ phase increase with pressure, similar to that of *β*-B. Based on an empirical relation, the hardness of *m*-B_16_ and *o*-B_16_ is calculated to be about 56 GPa and 61 GPa, respectively, approaching that of the *α*-Ga-B, suggesting all of them are potential superhard materials. The calculated electronic structures indicate that all these novel phases are metallic. In addition, the difference charge density maps reveal the accumulations of electrons into the interstitial sites of the lattice may play an important role under high pressure. From the crystal orbital Hamilton population (COHP) analysis one find that the *o*-B_24_ phase has stronger bonds than that of the *c*-B_56_ phase and the interaction changes during the *m*-B_16_ phase transforming to the *o*-B_16_ phase. The present work strongly suggests further study is needed to explore the behaviours of boron under high pressure conditions.

## Methods

Although evolutionary simulation method like USPEX^67^,^68^,^69^ have been adopted in successfully predicting potential crystal structures of Boron, all new structures studied in this work were conceived and constructed by hand, which is activated by Chaoyu He′s work^31^. The details of procedures in arriving these structure are described in the supplementary file.

For each crystal structure, the structural relaxations and electronic properties calculations were performed in the framework of density functional theory^70^, as carried out within the Vienna *ab initio* simulation package (VASP)^71^,^72^, with the projector augmented wave (PAW) method^73^. The 2s^2^2p^1^ electrons were treated as valence electrons. The generalized gradient approximation with the Perdew-Burke-Ernzerhof (PBE) functional^74^ for the exchange correlation was employed. A plane-wave basis with a cutoff energy of 500 eV was used to expand the wave functions. The *k*-point samplings in the Brillouin zone are 8 × 8 × 8 for *o*-B_24_, 7 × 7 × 7 for *c*-B_56_ and 4 × 14 × 14 for m-B_16_ based on the Monkhorst-Pack method. To ensure that the obtained structures are dynamically stable, phonon frequencies were calculated throughout the Brillouin zone using the phonon package^37^ with the forces calculated from VASP. The reliability of the pseudopotential approach has also been confirmed by the full-potential linearized augmented plane waves approach. The calculation of the elastic constants by the strain-stress relations was carried out using the CASTEP code^38^.

Some techniques such as the crystal orbital overlap population (COOP)^63^,^64^ and its analogous crystal orbital Hamilton population (COHP)^65^,^66^ can provide a straightforward view onto oribital-pair interactions, based on these techniques, one can analyze and interpret the bonding situation in solid-state materials. In the present work, to elucidate the bonding information in these four new boron phases, we adopted a variant of the familiar COHP approach that stems from a PW calculation and was dubbed “projected COHP” (pCOHP)^75^,^76^. In this approach, all the projection and analytic methods have been implemented in a standalone computer program which processes PAW parameters and self-consistent results from VASP.

## Supplementary Material

Supplementary Information Phase transitions, mechanical properties and electronic structures of novel boron phases under high-pressure-supp

## Figures and Tables

**Table 1:** Optimized Crystallographic Data of *o*-B_24_

Space group	*a* (Å)	*b* (Å)	*c* (Å)	*V* (Å^3^)	*ρ* (g/cm^3^)
*Pmna* (no. 53)	4.84	5.33	7.52	194.06	2.22

atom	position	*x*	*y*	*z*
B1	8*i*	0.6992	0.3584	0.9343
B2	8*i*	0.8236	0.3400	0.1512
B3	4*h*	0.5000	0.6347	0.3040
B4	4*h*	0.5000	0.8410	0.4915

**Table 2:** Optimized Crystallographic Data of *c*-B_56_

Space group	*a* (Å)	*b* (Å)	*c* (Å)	*V* (Å^3^)	*ρ* (g/cm^3^)
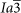 (no. 206)	7.62	7.62	7.62	443.08	2.27

atom	position	*x*	*y*	*z*
B1	48*e*	0.3482	0.5632	−0.3420
B2	8*a*	0.5000	−0.5000	−0.5000

**Table 3:** Optimized Crystallographic Data of *m*-B_16_

Space group	*a* (Å)	*b* (Å)	*c* (Å)	*β*	*V* (Å^3^)	*ρ* (g/cm^3^)
*C2/c* (no. 15)	11.04	3.19	3.13	74.62°	106.14	2.71

atom	position	*x*	*y*	*z*
B1	8*f*	0.0779	0.1238	0.7440
B2	8*f*	0.1736	0.3582	0.4642

**Table 4:** Optimized Crystallographic Data of *o*-B_16_

Space group	*a* (Å)	*b* (Å)	*c* (Å)	*V* (Å^3^)	*ρ* (g/cm^3^)
*Imma* (no. 74)	10.62	2.96	3.28	102.92	2.79

atom	position	*x*	*y*	*z*
B1	8*i*	0.0813	0.2500	0.1520
B2	8*i*	0.1718	0.2500	0.3409

**Table 5:** Calculated values of elastic constants *C_ij_* (GPa) of the newly discovered phases

Structure	*C*_11_	*C*_22_	*C*_33_	*C*_44_	*C*_55_	*C*_66_	*C*_12_	*C*_13_	*C*_15_	*C*_23_	*C*_25_	*C*_35_	*C*_46_
*o*-B_24_	448	330	517	70	132	66	4	70	-	25	-	-	-
*c*-B_56_	417	-	-	160	-	-	95	-	-	-	-	-	-
*m*-B_16_	655	499	590	277	277	234	26	66	81	91	3	61	19
*o*-B_16_	731	683	614	331	237	249	46	18	-	61	-	-	-

**Table 6:** *B_0_* (GPa) (is the isothermal bulk modulus, obtained by fitting a third-order Birth-Murnaghan equation of state), *B_0_*′, *V_0_* (Å^3^/f.u.), bulk modulus *B* (GPa) (is the adiabatic bulk modulus, obtained by the DFT calculation), shear modulus *G* (GPa), Young′s modulus *E* (GPa), Poisson′s ratio *σ* and *B*/*G* ratio for the whole structure types of boron at 0 GPa and 0 K

	*α*-B	*β*-B	*γ*-B	α*-B	*α*-Ga-B	*o*-B_24_	*c*-B_56_	*m*-B_16_	*o-*B_16_
*B_0_*	213	199	222	209	262	158	204	226	-
*B_0_*′	3.7	3.6	3.6	3.7	3.6	3.4	3.4	3.5	-
*σ*	0.13	0.19	0.10	0.15	0.09	0.20	0.19	0.09	0.09
*E*	468	367	527	437	648	286	380	563	625
*B*	210	197	222	207	263	161	203	229	252
*G*	207	154	239	191	297	119	160	258	287
*B*/*G*	1.01	1.27	0.93	1.09	0.89	1.35	1.26	0.89	0.88

**Table 7:** The calculated hardness values *H_v_* (GPa) for the related structures of boron from the empirical scheme (*H_v_^a^*) and Lyakhov-Oganov method(*H_v_^b^*)

	*α*-B	*β*-B	*γ*-B	α*-B	*α*-Ga-B	*o*-B_24_	*c*-B_56_	*m*-B_16_	*o-*B_16_
*H_v_^a^*	41.7	25.8	50.6	36.2	61.4	20.0	26.6	56.2	60.7
*H_v_^b^*	31.2	29.2	37.7	35.3	48.5	33.7	36.1	45.9	46.2
*Ref.*	42^a^, 38.96^d^	45^b^, 25 ~ 30^e^	50^c^, 48.8^f^	36.96^d^	-	-	-	-	-

^a^Ref. 41. ^b^Ref. 42. ^c^Ref. 7. ^d^Ref. 31. ^e^Ref. 17. ^f^Ref. 48.
