# Supplementary material for: Phase transitions, mechanical properties and electronic structures of novel boron phases under high-pressure: A first-principles study
Source: Sci Rep. 2014 Oct 27;4:6786. doi: 10.1038/srep06786 (PMC5381373; doi:10.1038/srep06786)
Supplement: Supplementary Information — Phase transitions, mechanical properties and electronic structures of novel boron phases under high-pressure-supp [file srep06786-s1.pdf]

Phase transitions, mechanical properties and electronic structures of novel boron phases under high-pressure: A first-principles study

Changzeng Fan[*,†], Jian Li[†], Limin Wang[†]
[†]State Key Laboratory of Metastable Materials Science and Technology, Yanshan University, Qinhuangdao 066004, China

Ⅰ.The Methods used for Constructing These Structures

In present work, we construct three new boron structures by hand with the methods as discussed in the following. Three simple but very useful strategies are introduced for generating these boron structures. *Strategy one*: each icosahedron is considered as an entity and then be placed into high symmetry positions of a cuboid lattice. *Strategy two*: a full sp^2 type cubic carbon structure is selected. Then all carbon atoms are replaced by the boron atoms, followed by placing more boron atoms on high symmetry positions of the cubic structure in order to satisfy a reasonable coordinates for boron atoms. *Strategy three*: a very simple two dimensional boron layers are conceived and then be placed into a monoclinic lattice with its partners by mirror symmetry and then be compressed along the direction perpendicular to the two dimensional plane, in the purpose of fulfilling a reasonable coordinates for boron atoms.

The procedure of implementing each idea is illustrated in the following figures (Fig. S1-S3).

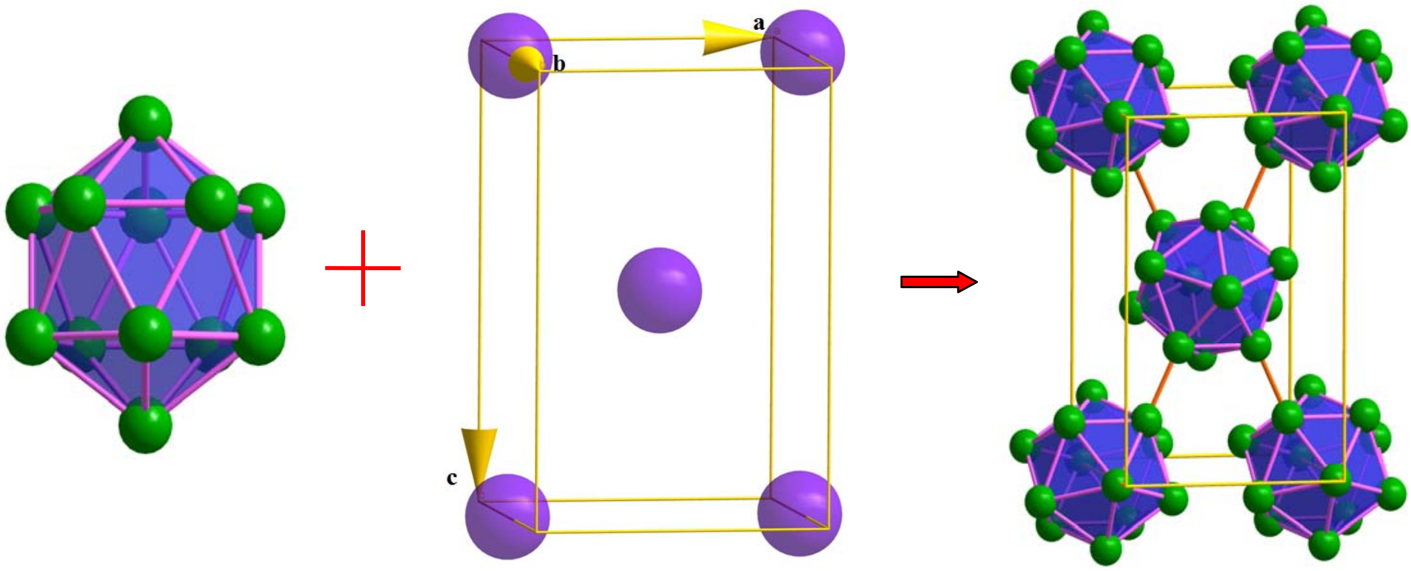

Figure S1 | Procedure for constructing the *o* -B_{24} phase.

As shown in Fig. S1, one can obtain the *o* -B_{24} phase in the following four simple steps. (1) Construct an icosahedron as observed in α-Boron. (2) Construct a simple cuboid lattice. (3) Insert the icosahedra into the centre of the cuboid lattice and the middle points of the four edges along the *b* direction as shown in the middle panel of Fig. S1. (4) Adjudge the lattice constants *a*, *b* and *c* of the cuboid lattice to make the calculated the inter-icosahedral bond lengths be reasonable.

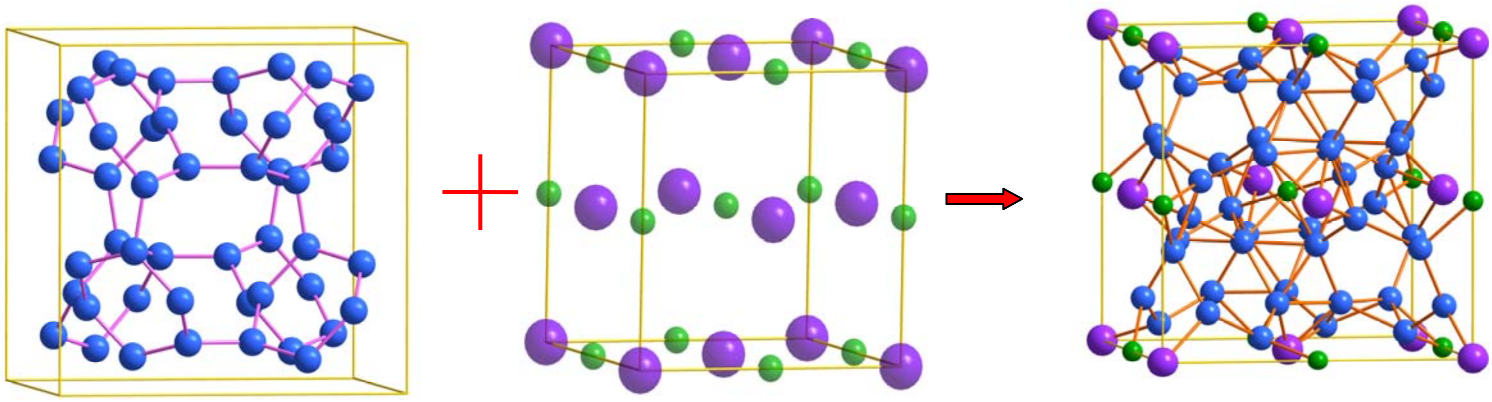

(A) (B) (C)

Figure S2 | Procedure for constructing the *c*-B_{56} phase.

A full sp^2 type carbon phase (S.G. 206) reported by Chaoyu He *et.al.*[1] has been selected as a protocol to build up the boron structure, as shown in Fig.S2A. Such sp^2-diamond structure[2] was also proposed as the 3/6/*c*3 and 3/6/*c*5 configurations by E. Koch *et.al* in 1995,[3] which contains only one inequivalent atomic position at the Wyckoff position of 48*e* (0.888,0.985,0.155). Based on this protocol, all initial cabon atoms are replaced by the boron atoms firstly. Then additional boron atoms placed on high symmetry positions of the lattice are added, as plotted in Fig.S2B. Finally, the new cubic boron structure are obtained by fully relaxing the atoms positions during geometry optimization by first-principles calculations – see Fig.S2C.

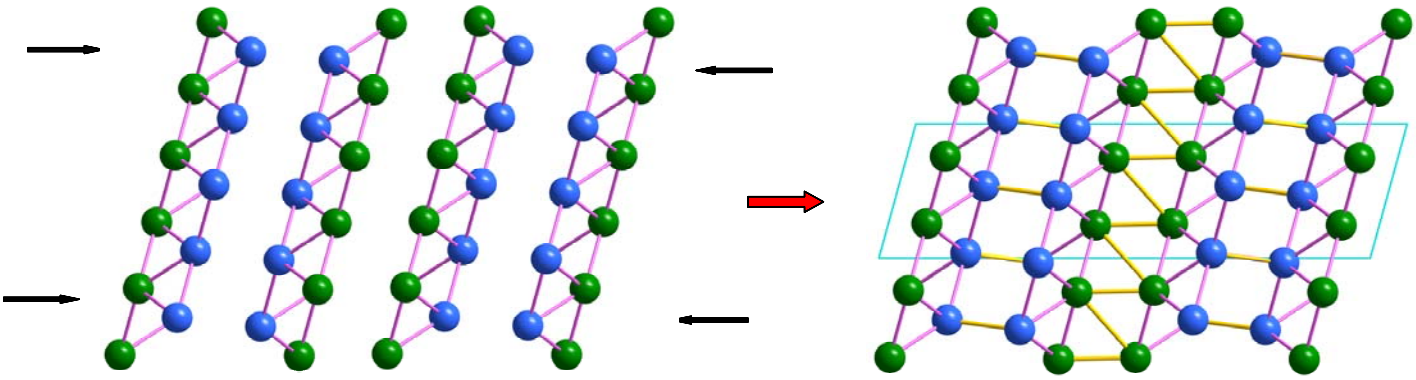

Figure S3 | Procedure for constructing the *m*-B_{16} phase. Green and blue spheres represent B atoms with different locations (B1 and B2 atoms), respectively.

Firstly, a novel two-dimensional (2D) boron structure was conceived that composed of triangular and rhombic motifs. Schematic structure of the 2D boron projected along the [010] direction is plotted in the left panel of Fig.S3. Secondly, we regard the 2D boron structure as a layer of boron atoms and such layer with its partner by mirror symmetry are arranged parallel according to the order as shown in the left panel of Fig.S3. Finally, a novel three-dimensional (3D) boron structure are obtained by compressing the periodic stacked 2D layers along the direction perpendicular to the two dimensional plane, until a reasonable inter-layer distance and reasonable coordinates for boron atoms are satisfied by first-principles calculations geometry optimization.

II. Stabilities of New High-Pressure Phases

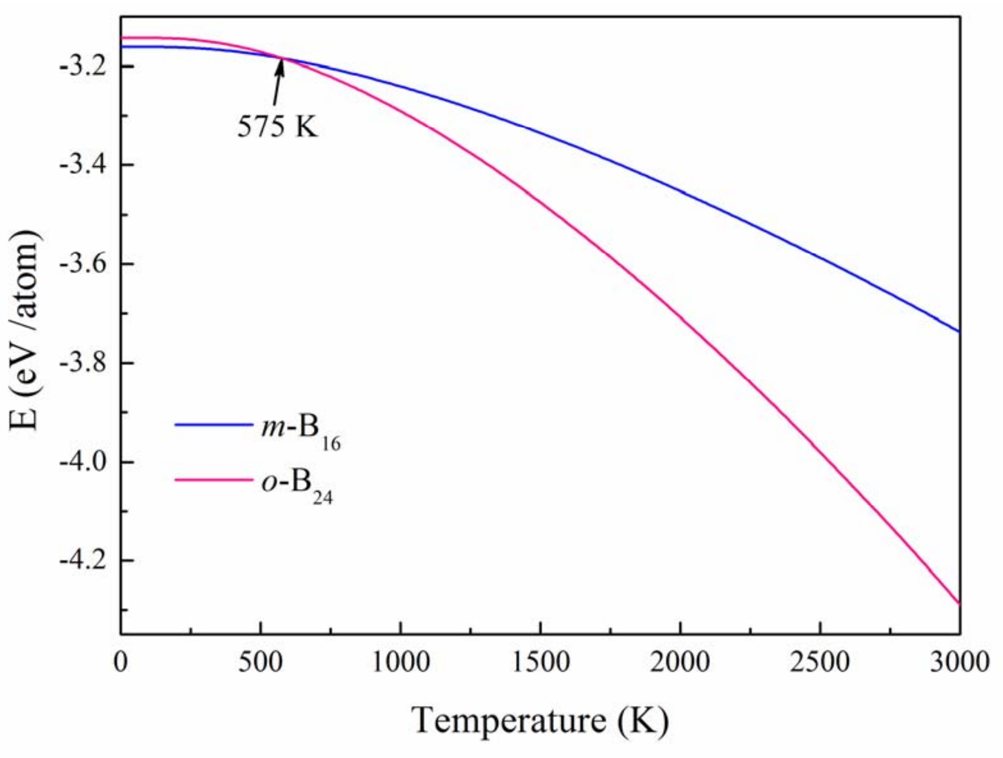

Figure S4│ The calculated energy curves as a function of temperature for the *o*-B_{24} and *m*-B_{16} phase.

III. Tables

Table S1 | The intra- and inter-icosahedral B-B bond types, bond lengths *d* and numbers for the *o*-B_{24} phase (B_1~B_4 means different boron atoms as shown in Fig.1 and B_1-B_2/B_1-B_2' means there are two inequivalent bonds between B_1 and B_2 atoms.)

		Intra-icosahedron			
Bond	*d* (Å)	Numbers	Bond	*d* (Å)	Numbers
B_1-B_1	1.81	2	B_2-B_3	1.82	4
B_1-B_2	1.75	4	B_2-B_4	1.76	4
B_1-B_3	1.76	4	B_3-B_4	1.79	2
B_1-B_4	1.86	4	B_1-B_2'	1.84	4
B_2-B_2	1.71	2			

		Inter-icosahedron			
Bond	*d* (Å)	Numbers	Bond	*d* (Å)	Numbers
B_1-B_1	1.92	4	B_1-B_3	2.04	8
B_2-B_2	1.65	4	B_4-B_4	1.70	2

Table S2 | The intra- and inter-cube bond types and bond lengths *d* for the *c*-B_{56} phase (B_1 and B_2 mean different boron atoms, B_1-B_1/B_1-B_1'/B_1-B_1'' means there are three inequivalent bonds between B_1 and B_1 atoms).

		Intra-cube			
Bond	*d* (Å)	Numbers	Bond	*d* (Å)	Numbers
B_1-B_1	1.70	2	B_1-B_1'	1.76	4
B_1-B_2	1.74	12	B_1-B_1''	1.82	10
		Inter-cube			
Bond	*d* (Å)	Numbers	Bond	*d* (Å)	Numbers
B_1-B_1	1.70	6	B_1-B_1'	1.76	12

Table S3 | The intra- and inter-layer bond types and bond lengths *d* for the *m*-B_{16} phase (B_1 and B_2 mean different boron atoms, B_1-B_2/B_1-B_2'/ B_1-B_2''/B_1-B_2'''means there are four inequivalent bonds between B_1 and B_2 atoms).

	Intra-layer		
Bond	*d* (Å)	Bond	*d* (Å)
B_1-B_1	1.76	B_1-B_2'	1.89
B_1-B_2	1.71	B_1-B_2''	1.98
B_2-B_2	1.81	B_1-B_2'''	2.34
	**Inter-layer**		
Bond	*d* (Å)	Bond	*d* (Å)
B_1-B_1	1.73	B_1-B_1'	2.13
B_2-B_2	1.78		

Table S4 | The intra- and inter-layer bond types and bond lengths d for the o-B_{16} phase(B_1 and B_2 mean different boron atoms, B_1-B_1/B_1-B_1' means there are two inequivalent bonds between B_1 and B_1 atoms and B_1-B_2/B_1-B_2'/ B_1-B_2'' means there are three inequivalent bonds between B_1 and B_2 atoms).

	Intra-layer		
Bond	d (Å)	Bond	d (Å)
B_1-B_1	1.78	B_1-B_2'	1.88
B_1-B_2	1.87	B_1-B_2''	1.92
B_2-B_2	1.81		
	Inter-layer		
Bond	d (Å)	Bond	d (Å)
B_1-B_1	1.73	B_1-B_1'	2.48
B_2-B_2	1.77		

IV. Reference

(1) He, C. Y., Sun, L. Z., Zhang, C. X. & Zhong, J. X. Systematic enumeration of crystalline networks with only sp^2 configuration in cubic lattices. *arXiv*:1302.6430.

(2) He, C. Y., Sun, L. Z., Zhang, C. X. & Zhong, J. X. Two viable three-dimensional carbon semiconductors with an entirely sp^2 configuration. *Phys. Chem. Phys. Chem.* **15**, 680-684 (2013).

(3) Koch, E. & Fischer, W. Sphere packing with three contacts per sphere and the problem of the least dense sphere packing. *Zeitschrift für Kristallographie* **210**, 407-414 (1995).
